# Tobacco cessation services for cancer survivors: A capture and coding of U.S. National Cancer Institute (NCI)-Designated Cancer Center webpages

**DOI:** 10.1093/ntr/ntag093

**Published:** 2026-04-24

**Authors:** Karissa Tran, Leeann N Siegel, Madina Smagulova, Grace C Huang, Molly Frauenholz, Lindsey Kirshner, Heather D’Angelo, Susan Czajkowski, Emily Tonorezos, Stephanie R Land

**Affiliations:** Department of Psychology, University of California, Los Angeles, CA 90095, United States; Center for Communication and Media Impact, RTI International, Durham, NC 27713, United States; Public Health Practice, Westat, Bethesda, MD 20814, United States; Public Health Practice, Westat, Bethesda, MD 20814, United States; Division of Cancer Control and Population Sciences, National Cancer Institute, Rockville, MD 20850, United States; Clinical Research, Westat, Bethesda, MD 20814, United States; Division of Cancer Control and Population Sciences, National Cancer Institute, Rockville, MD 20850, United States; Division of Cancer Control and Population Sciences, National Cancer Institute, Rockville, MD 20850, United States; Division of Cancer Control and Population Sciences, National Cancer Institute, Rockville, MD 20850, United States; Division of Cancer Control and Population Sciences, National Cancer Institute, Rockville, MD 20850, United States

**Keywords:** cancer center, National Cancer Institute, smoking, smoking cessation, tobacco, tobacco dependence, tobacco treatment, tobacco use

## Abstract

**Introduction:**

People who continue to smoke after receiving a cancer diagnosis experience adverse outcomes. National Cancer Institute-Designated Cancer Centers (NCI-DCCs) can help prevent these adverse outcomes by offering tobacco cessation services. This study examined the online availability of tobacco cessation service information by searching NCI-DCC websites to identify webpages offering tobacco treatment services and/or resources (“web capture”) and by systematically coding these webpages to characterize the services offered and information provided (“webpage coding”).

**Methods:**

The web capture was conducted during 2023 for 65/72 NCI-DCCs (excluding 7 Basic Laboratory NCI-DCCs) by (1) entering key terms related to tobacco cessation into the search box on the homepages, (2) reviewing the homepages, and (3) reviewing drop-down menus. The webpage coding was conducted during 2024 and 2025 using a multiple-coder protocol designed by NCI subject matter experts and was refined iteratively over three rounds of pilot testing.

**Results:**

A total of 225 webpages offered tobacco cessation services. NCI-DCCs had from 0 to 19 webpages captured. Most NCI-DCCs offer counseling services (72.3%), medication (66.2%), quitlines (69.2%), and external resources and services/programs (66.2%). Less commonly, NCI-DCCs offer support groups, group classes, and/or workshops separate from counseling services (35.4%); and NCI-sponsored cessation programs (53.8%).

**Conclusions:**

The results suggest that many NCI-DCCs offer tobacco cessation services; however, there is still room for improvement in the availability and promotion of tobacco cessation services for cancer survivors.

**Implications:**

National Cancer Institute-Designated Cancer Centers (NCI-DCCs) offer tobacco cessation services to prevent adverse outcomes among cancer survivors. By searching NCI-DCC websites to identify webpages offering tobacco treatment services and/or resources and characterizing services and information, this study examined the online availability of tobacco cessation service information. Most NCI-DCCs offer counseling, medication, quitlines, and external resources and services/programs. However, support groups, group classes, and/or workshops separate from counseling and NCI-sponsored cessation programs are less common. Findings inform providers and policy-makers of the remaining need for smoking cessation services in cancer care settings and to ensure that patients are informed about such services via cancer center websites.

## Introduction

The leading preventable cause of cancer and death from cancer is tobacco use. In the United States, smoking is responsible for ~20% of all new cancer diagnoses and 30% of all cancer deaths. ^1-3^ Cancers caused by smoking include cancers of the lung, larynx, oral cavity and pharynx, esophagus, pancreas, bladder, stomach, colon and rectum, liver, cervix, and kidney, and acute myeloid leukemia.^4^^,^^5^ Cancer patients who continue to smoke after receiving a diagnosis experience adverse outcomes, including but not limited to decreased survival rates, worse responses to cancer treatment, a greater likelihood of developing a second primary cancer, poor quality of life, and an increased total symptom burden relative to those who quit smoking.^6-10^ Despite this, many cancer patients continue to smoke after diagnosis. In 2023, 11.4% of U.S. adult cancer survivors currently smoked.^11^

Effective tobacco cessation interventions exist, including pharmacotherapy (eg, bupropion, varenicline, nicotine replacement therapy) and behavioral counseling (eg, individual counseling, group counseling).^12-14^ These interventions can be administered individually or in combination, although they tend to be more effective when administered in combination.^15^^,^^16^ The National Comprehensive Cancer Network (NCCN) recommends that pharmacotherapy and behavioral counseling be administered in combination for people who have been diagnosed with cancer and are currently smoking.^17^

NCI-Designated Cancer Centers (NCI-DCCs) are a nationwide set of cancer treatment facilities that have met rigorous standards with respect to laboratory and clinical research. Day *et al.* conducted online website audits in 2017 and found that 15 NCI-DCCs (24.2% of the 62 centers studied) did not have system-wide tobacco cessation treatment programs, whereas 47 NCI-DCCs (76%) did have system-wide tobacco cessation treatment programs. They also conducted a systematic literature review and found that 36% of identified tobacco cessation treatment programs were not listed anywhere on the websites of the NCI-DCCs.^18^ The Cancer Center Cessation Initiative (C3I) was launched in 2017 to increase the availability of tobacco cessation interventions, like pharmacotherapy and behavioral counseling, at NCI-DCCs.^19^^,^^20^ The initiative provided funding for 52 centers in three rounds, 2017, 2018, and 2020.

As of 2023, there were 72 NCI-DCCs, of which 7 were Basic Laboratory Cancer Centers, primarily focused on laboratory research. The remaining 65 delivered advanced cancer therapy to patients throughout broad catchment areas. Recently, Littman *et al.* examined the websites of 64 NCI-DCCs to assess the availability and quality of actionable smoking cessation information, such as information that serves to educate patients about smoking cessation treatment and strategies or about the harms of smoking and benefits of quitting (for example, “smoking cessation after cancer diagnosis is associated with decreased mortality”). They found that 55% of websites hosted cessation educational materials.^21^ By examining the availability and quality of educational materials on the websites of NCI-DCCs, Littman and colleagues were able to assess the utility of these websites as smoking cessation interventions, drawing conclusions about how educational materials available on the websites themselves may affect patients who smoke. The present study is focused on a complementary question: what can we learn from NCI-DCCs’ websites about tobacco cessation services available to their patients in-person, online, or in other formats? This includes questions such as: do they offer services such as counseling and pharmacotherapy? Are services provided in-house or externally? With what frequency do they offer these services? Therefore, the first aim of the present study is to conduct an online web capture to identify relevant webpages on NCI-DCC websites offering tobacco treatment services and/or resources (ie, “web capture” phase). The second aim is to systematically code NCI-DCC websites and affiliated websites to characterize key features of programs offered and information provided (ie, “webpage coding” phase). The information gained will be essential for developing targeted programs, information, and resources to accelerate tobacco cessation efforts at NCI-DCCs in the future. This effort is complementary to the work by Littman *et al.* in that our focus is on assessing, via information provided on NCI-DCC websites, the types of smoking cessation services that are being offered at NCI-DCCs.

## Methods

### “Web capture” phase

An online web capture was conducted by one author (KT) from June 2023 to November 2023 on 65 NCI-DCC websites (after excluding 7 Basic Laboratory NCI-DCCs from the 72 NCI-DCCs that existed in 2023) to identify webpages offering tobacco treatment services and/or tobacco treatment resources.

NCI-DCC websites were identified through https://www.cancer.gov/research/infrastructure/cancer-centers/find. Webpages to be captured were identified in three ways. First, the search box on the homepage of NCI-DCC websites was used to obtain search box results. Three separate searches were conducted with the keywords “Tobacco Cessation,” “Tobacco,” and “Smoking.” All search box results were reviewed for the first 41 NCI-DCC websites examined. To improve the efficiency of the webpage capture for the remaining NCI-DCC websites examined, only the top 10 results belonging to the cancer center were reviewed. The top 10 results are displayed on the first page for most cancer centers. Most individuals are unlikely to search beyond the first page of results.^22^ Webpages that mentioned the words “tobacco cessation program” without providing a link or additional information were not captured. The rationale for not capturing such webpages is that without a link or additional information, the webpage does not give information about what services are available to cancer center patients. Second, the homepage of each NCI-DCC website was examined. Third, all tabs (ie, drop-down menus) on NCI-DCC websites were reviewed. Primary tabs and embedded secondary tabs were clicked through following the hierarchical organization of pages. When information in a given tab was organized by a long list of cancer types, only “lung cancer” or “thoracic cancer” was reviewed, given that cigarette smoking is known to be the leading risk factor for lung cancer.^23^ If the webpage was captured, it was moved on to the coding phase. [See Supplemental Appendix for technical definitions of internet-related terminology.]

It is necessary in a web content review to include criteria on whether to further follow hyperlinks from a given webpage or to stop at the given webpage. The criterion for the present study was whether the webpage was cancer-specific (in which case, hyperlinks found in the webpage were followed) or not cancer-specific (in which case, hyperlinks found in the webpage were not followed). “Cancer-specific” webpages were those within the cancer center domain (eg, https://www.mdanderson.org/) or within an affiliated health system or university or other affiliated institution domain where one of the following conditions was met: (1) described tobacco treatment services provided by the cancer center, (2) had cancer center branding, or (3) included text directed to cancer patients. If one of these three requirements was met, hyperlinks on the webpage could be further followed (ie, capture the given webpage and potentially capture additional webpages via hyperlinks on the given webpage). If the webpage was not “cancer-specific” and contained information on tobacco treatment services and/or resources from an affiliated health system or university or other affiliated institution domain, hyperlinks were not further followed. (The given webpage was captured, but the search did not follow hyperlinks on the given webpage.)

### “Webpage coding” phase

#### Codebook development

The codebook was designed by the authors (NCI subject-matter experts) and was refined iteratively between November 2023 and June 2024 over three rounds of pilot-testing. A total of 12 NCI-DCCs (3, 3, and 6 NCI-DCCs, respectively) were used in the pilot-testing phase. During each round of testing, two to three team members coded NCI-DCCs independently, compared codes, and resolved discrepancies through group discussion. The pilot-testing phase was further used for consensus-building and developing inter-coder reliability among team members. Each pilot-testing round yielded new decision rules that were folded into the codebook for added clarity, and cancer centers were recoded accordingly to reflect the codebook updates. Table 1 lists the codes used for the webpage coding phase.

**Table 1:** Codes used for the webpage coding phase.

Item	Codes
Section I. General Information About the Webpage	
1.2. Categorize the domain of the webpage. (Select one.)	1. Cancer center 2. Affiliated hospital, health system, or university domain that is specific to cancer patients 3. Affiliated hospital, health system, or university domain that is *not* specific to cancer patients 4. Something else (explain) 5. Not applicable
1.3. What smoking cessation information/services are observed on the webpage? (Select all that apply.)	1. Counseling services (eg, in person, phone) 2. Medication (eg, prescription, nicotine replacement therapy) 3. Support groups, group classes, workshops (separate from counseling) 4. Quitline 5. NCI-sponsored programs (eg, Smokefree.gov, SmokefreeTXT, other similar programs) 6. Educational resource (eg, information about quitting, links to internal resources, frequently asked questions, webinar, informational videos, blogs, newsletters) 7. Links to other external resources and services/programs that are not provided by this health system or cancer center (eg, American Cancer Society) 8. Other (specify)
1.4. Is there any mention or description of smoking cessation information or services offered in languages other than English? (Select one.)	1. Yes (describe) 2. No 3. Not applicable
1.5. Name of the tobacco treatment program.	Insert a specific proper noun for the tobacco cessation program or else write "None."
1.6. Eligibility for services mentioned. (Select all that apply.)	1. Cancer center patients 2. Patients of the affiliated hospital or health system 3. Cancer patients 4. Caregivers 5. General public/community members 6. Not specified 7. Other (specify)
1.7. What are the credentials of staff members who will provide any of the cessation services? (Select all that apply.)	1. Tobacco treatment specialist (TTS) 2. Psychologist/therapist (Ph.D., M.S.W.) 3. Clinician (eg, Nurse, R.N., doctor, M.D.) 4. Pharmacist 5. Not specified 6. Other (specify)
Section II. Counseling Service Details (For all items, “select all that apply”)	
2.1. Who runs the counseling service/program?	1. Cancer center 2. Affiliate institution of cancer center 3. Other organization partnering with the cancer center 4. Not specified
2.2. Counseling format: What type of counseling is mentioned?	1. In-person 2. Phone call or video call (eg, telehealth) 3. Individual 4. Group counseling 5. Digital/mobile health (eg, text messaging, app, chatbot; no real-time counselor) 6. Consult/brief counseling 7. Not specified
2.3. Counseling cost-related information mentioned.	1. Free 2. Out-of-pocket 3. Insurance accepted 4. Not specified 5. Other (specify) [TEXT]
Section III. Medication Service Details (For all items, “select all that apply”)	
3.1. Format of medication services.	1. Participant is given the medication or a prescription in hand 2. Participant is mailed the medication 3. Participant is mailed a prescription 4. Prescription is electronically submitted to the pharmacy 5. Not specified 6. Other (specify)
3.2. Medication cost-related information mentioned.	1. Free 2. Out-of-pocket 3. Insurance accepted 4. Not specified 5. Other (specify)

#### Webpage coding

The captured webpages were coded by the same three coders (authors MS, MF, and LK) from August 2024 to January 2025. The webpages were coded by one person, and each result was verified in detail by a second person as part of a quality-control process. Discrepancies between the initial coder and the verifying reviewer were resolved through discussion and consensus. In instances where coding discrepancies could not be resolved, the study’s Principal Investigator (SL) served as the arbitrator of the final decision. Each captured webpage was again screened for relevance based on criteria established in the codebook—that is, removing duplicate webpages, webpages that no longer existed, or webpages that were entirely external (not associated with the cancer center or its institutional affiliates) or did not include information about cessation services provided. Following this effort, for those webpages considered relevant, coders classified the information provided on the webpage into one or more types of information or services (ie, counseling services, medication, support groups/group classes/workshops, quitlines, NCI-sponsored programs, external resources and services/programs). Moreover, the coding would be considered complete if the page only included information about quitlines, NCI-sponsored programs, or external resources and services/programs. Counseling services, medication, and support groups/group classes/workshops were only coded further if they were offered by the cancer center and/or an affiliated or partnering organization. If counseling services, medication, or support groups/group classes/workshops were found, coders proceeded to document additional information about these services (eg, eligibility, staff credentials, the entity providing the service, format, cost) based on codebook specifications. See Figure 1.

**Figure 1:**
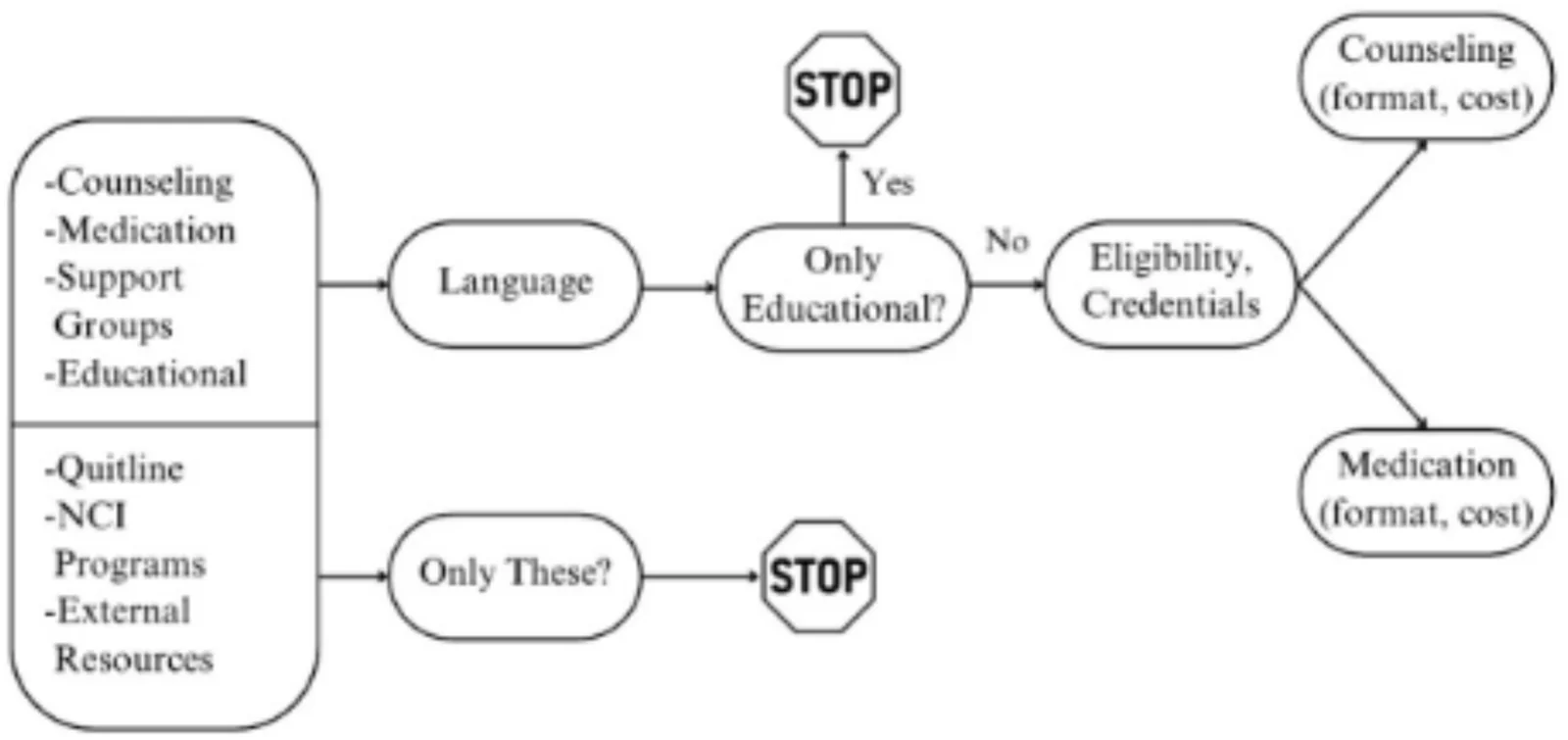
A graphic depiction of the webpage coding phase procedure.

### Statistical analysis

Data were collected at the webpage level. However, flag/composite variables were created at the cancer center level to indicate the presence or absence of each information or service and the cumulative number of identified services. Descriptive statistics (ie, frequencies, percentages) are provided at the cancer center level for clarity and ease of interpretation. Data were analyzed with the Statistical Package for the Social Sciences (SPSS).^24^

## Results

Associated with the 65 NCI-DCCs, 598 webpages were initially captured as potentially relevant. Of these, 225 (37.6%) NCI-DCC webpages were deemed relevant and were therefore coded, with a range between 0 and 19 per NCI-DCC and a median of 2. Eleven NCI-DCCs (16.9%) had no relevant webpages for coding; that is, for 11 NCI-DCCs, there was no webpage captured that provided information about tobacco cessation services available to NCI-DCC patients.

Webpage coding revealed that support groups/ group classes/workshops separate from counseling services were offered on the websites of 23/65 (35.4%) NCI-DCCs. Quitlines were mentioned on the websites of 45/65 (69.2%) NCI-DCCs. External resources and services/programs not provided by the health system or cancer center were offered on the websites of 43/65 (66.2%) NCI-DCCs. NCI-sponsored programs (eg, Smokefree.gov, SmokefreeTXT) were offered on the websites of 35/65 (53.8%) NCI-DCCs. Counseling services were offered on the websites of 47/65 (72.3%) NCI-DCCs—25/47 (53.2%) offered in-person counseling, 29/47 (61.7%) offered counseling via phone or video call (eg, telehealth), 27/47 (57.4%) offered individual counseling, 12/47 (25.5%) offered group counseling, 5/47 (10.6%) offered digital/mobile health counseling (eg, text messaging, app, chatbot; no real-time counselor), and 12/47 (25.5%) offered consult/brief counseling. Of the 47 NCI-DCCs that offered counseling services, 19/47 (40.4%) offered at least one counseling service for free. Of the same 47 NCI-DCCs, counseling services were provided by the cancer center at 38/47 (80.9%) NCI-DCCs, an affiliate institution of the cancer center at 21/47 (44.7%) NCI-DCCs, other organizations partnering with the cancer center at 5/47 (10.6%) NCI-DCCs, and an unspecified source at 1/47 (2.1%) NCI-DCC. Medication was offered by 43/65 (66.2%) NCI-DCCs. Of the NCI-DCCs that offered medication, 12/43 (27.9%) offered at least one medication service for free.

Additionally, webpage coding revealed that 16/65 (24.6%) NCI-DCCs offered tobacco cessation information or services in languages other than English. Eligibility for services specified cancer center patients at 34/65 (52.3%) NCI-DCCs, cancer patients more broadly at 4/65 (6.2%) NCI-DCCs, and patients of the affiliated hospital or health system at 14/65 (21.5%) NCI-DCCs. Caregivers were eligible for services at 5/65 (7.7%) NCI-DCCs. General public/community members were eligible for services at 18/65 (27.7%) NCI-DCCs. Eligibility for services was unspecified at 18/65 (27.7%) NCI-DCCs. Services were provided by tobacco treatment specialists at 26/65 (40%) NCI-DCCs, psychologists/therapists (eg, Ph.D., M.S.W.) at 10/65 (15.4%) NCI-DCCs, clinicians (eg, nurse, R.N., doctor, M.D.) at 20/65 (30.8%) NCI-DCCs, and pharmacists at 2/65 (3.1%) NCI-DCCs. The credentials of the staff who provided services were unspecified at 34/65 (52.3%) NCI-DCCs. See Table 2 for a summary of results.

## Discussion

The present study sought to quantify and categorize the tobacco cessation services advertised on cancer center websites. Of the 65 NCI-DCC websites reviewed, 54/65 (83.1%) provided information about tobacco treatment services or resources. NCCN recommends that pharmacotherapy and behavioral counseling be administered in combination for people who have been diagnosed with cancer and are currently smoking.^17^ We found that 47/65 (72.3%) NCI-DCCs listed counseling services, 43/65 (66.2%) NCI-DCCs listed medication, and 42/65 (64.6%) NCI-DCCs listed both counseling services and medication on their websites. Thus, although C3I resulted in an increase in the availability of and referral to tobacco treatment services,^25^^,^^26^ our results do not reflect significant further progress as compared with the 2017 online audit by Day *et al*.^18^

Our findings differ from the complementary recent work by Littman and colleagues.^21^ Their search for smoking cessation educational information identified 35 NCI-DCCs that provided such information. Their work examined online information that serves as a cessation intervention in its own right, whereas the present study is focused on using webpages to understand services that are provided, such as medication and counseling. In addition to differences that may be due to varying search methodologies, these results may indicate that more cancer centers provide some type of service (identified in the present study) without necessarily providing patient online educational materials (the focus of the Littman study). Both cessation services and patient education about cessation are important.

**Table 2:** Cancer center-level coding results.

Content	n/N (%)
Support Groups/Group Classes/Workshops Separate From Counseling	23/65 (35.4%)
Quitlines	45/65 (69.2%)
External Resources and Services/Programs	43/65 (66.2%)
NCI-Sponsored Programs	35/65 (53.8%)
Counseling Services	47/65 (72.3%)
In-Person	25/47 (53.2%)
Phone/Video Call	29/47 (61.7%)
Individual Counseling	27/47 (57.4%)
Group Counseling	12/47 (25.5%)
Digital/Mobile Health Counseling	5/47 (10.6%)
Consult/Brief Counseling	12/47 (25.5%)
Cost of Counseling: Free	19/47 (40.4%)
Counseling Service Provider	
Cancer Center	38/47 (80.9%)
Affiliate Institution	21/47 (44.7%)
Other Organizations	5/47 (10.6%)
Unspecified Provider	1/47 (2.1%)
Medication	43/65 (66.2%)
Cost of Medication: Free	12/43 (27.9%)
Services in Languages Other Than English	16/65 (24.6%)
Eligibility for Services	
Cancer Patients at NCI-DCCs	34/65 (52.3%)
Cancer Patients Broadly	4/65 (6.2%)
Patients of the Affiliated Hospital or Health System	14/65 (21.5%)
Caregivers	5/65 (7.7%)
General Public/Community Members	18/65 (27.7%)
Unspecified	18/65 (27.7%)
Credentials of the Person Providing the Service	
Tobacco Treatment Specialists	26/65 (40%)
Psychologists/Therapists	10/65 (15.4%)
Clinicians	20/65 (30.8%)
Pharmacists	2/65 (3.1%)
Unspecified	34/65 (52.3%)

Our study identified room for improvement in the online presentation of tobacco cessation services and resources for patients, which may in turn indicate room for improvement in the provision of services. There may be a lack of awareness of the strong rationale for cancer centers to make smoking cessation services available to patients, a lack of familiarity with cessation interventions, or perceived barriers to the adoption and implementation of evidence-based interventions, strategies, and policies aimed at promoting healthy behaviors among cancer survivors. And yet the potential benefit for tobacco cessation as well as the effectiveness of interventions are well established.^27^ Indeed, the publication of NCI Monograph 23 on treating smoking in cancer patients curated evidence on the importance of this support and successful approaches.^27^ Furthermore, implementation strategies for smoking cessation in oncology settings are well-developed.^28^ Quitlines and external resources and services/programs, which were offered on the websites of 69.2% and 66.2% of NCI-DCCs (respectively), reduce institutional costs and relieve staff burdens.

It is important to highlight that this study is not intended to report on services “provided” by NCI-DCCs; rather, it is intended to report on services presented and made available to public consumers (ie, “findability” vs. “availability”).^29^ The information obtained from these web capture and webpage coding activities can be useful in devising further, more detailed efforts to assess the provision of services and barriers to such provision and pointing to areas of particular need within NCI-DCCs. We found that 11 NCI-DCCs (16.9%) had no relevant webpages for coding; therefore, future research should consider utilizing survey and informant-based assessments of NCI-DCCs (engaging NCI-DCC staff to provide this information), which could provide additional important details to target specific areas of need and potential barriers to service provisions.

One limitation of this study is that although we adopted a comprehensive and systematic approach for web capture and webpage coding, there may have been information or resources that we missed. Our capture and coding offer a snapshot of the resources mentioned on webpages and do not necessarily include all services, given the evolving nature of web content. Websites are updated regularly, and the information will likely have changed since our assessment.^29^ If extant services were not captured and coded in our study, this may suggest that patients will be less likely to become aware of the services, and websites might be improved. In addition, we recognize that websites are only one of the communication channels available at NCI-DCCs, and we do not know whether the presence of resources on the websites translated into patient uptake of those services.

Tobacco use cessation is a crucial strategy for patients diagnosed with cancer to use to improve their own prognosis and to potentially increase the efficacy of their tumor therapy. Indeed, tobacco cessation treatment has been deemed a “pillar” of cancer care.^30^ Many cancer treatment settings do provide support to patients for tobacco use cessation, yet the opportunity to further develop and promote tobacco treatment programs and resources remains.

## Supplementary Material

Appendix_clean_ntag093(1)

## Data Availability

Data without institutional identifiers will be made available at the time of publication at the NCI Cancer Control Data Repository (ccddr.cancer.gov). It will be publicly available without limitation.
