# Supplementary material for: Tobacco cessation services for cancer survivors: A capture and coding of U.S. National Cancer Institute (NCI)-Designated Cancer Center webpages
Source: Nicotine Tob Res. 2026 Apr 24;28(11):1855–61. doi: 10.1093/ntr/ntag093 (PMC13687891; doi:10.1093/ntr/ntag093)
Supplement: Supplementary file 1 [file appendix_clean_ntag093(1).docx]

Appendix Definitions

**First page (of search results)***: The "first page" of search engine results is the initial, highly curated page of links and information displayed by a search engine in response to a user's query.

**Homepage**:** The introductory page of a website, typically serving as a table of contents for the site. Note: In cases where the cancer center website was housed within a larger healthcare system or university domain, the cancer center’s main homepage (rather than the parent institution’s homepage) was treated as the homepage for web review purposes.

**Hyperlink (often simply called a "link")*:** A clickable element in a digital document or webpage that acts as a connector, directing the user to another location, such as a different webpage, a specific section on the same page, or an external file/resource.

**Search box***: User interface element, typically consisting of an input field and a submit button, that allows users to type in queries to find specific content within a website.

**Webpage***: A single document, written in languages like HTML, that displays content (text, images, videos, links) and is viewed in a web browser, forming part of a larger collection called a website.

***** Definitions provided by AI-assisted Google search.

** Definition provided by Oxford Languages.
